# Integrated physiological and transcriptomic analyses reveal cell wall remodeling underlying firmness retention in a firm-fruited blueberry genotype

**DOI:** 10.3389/fpls.2026.1962192

**Published:** 2026-10-08

**Authors:** Bowei Wang, Chenxin Yu, Yibo Ma, Xiaoxuan Tang, Yadong Li, Haiyue Sun

**Affiliations:** College of Horticulture, Jilin Agricultural University, Changchun, China

**Keywords:** blueberry, cell wall metabolism, firmness, postharvest texture, β-galactosidase

## Abstract

**Introduction:**

Fruit softening is a major factor limiting the postharvest quality, transportability, and shelf life of blueberry, but the physiological and molecular basis of firmness variation remains incompletely understood.

**Methods:**

The soft-fruited cultivar ‘Duke’ and the firm-fruited genotype ‘2017D’ were compared using phenotypic, physiological, histological, biochemical, transcriptomic, and transient functional analyses.

**Results:**

‘2017D’ maintained substantially higher firmness during fruit development, together with greater retention of several texture attributes, including chewiness, gumminess, resilience, and cohesiveness. Histological observations showed that ‘2017D’ retained a comparatively more continuous mesocarp organization at the blue stage. Cell wall analysis further revealed greater retention of structural polysaccharides and insoluble pectin in ‘2017D’, whereas ‘Duke’ accumulated more soluble pectin during the later developmental stages. Lower PG and β-galactosidase activities in ‘2017D’ were consistent with reduced cell wall polysaccharide degradation during ripening. RNA-seq analysis identified 6,168 differentially expressed genes between the two genotypes, with significant enrichment of pathways and biological processes associated with hormone signaling, carbohydrate metabolism, cell wall organization, and related ripening processes. Among the cell wall-related candidate genes, VcβGAL369 showed a pronounced expression difference between the two genotypes and a distinct developmental expression pattern and was therefore selected for further characterization. Transient silencing of VcβGAL369 increased fruit firmness and promoted cell wall component retention, whereas transient overexpression reduced fruit firmness and promoted cell wall disassembly.

**Discussion:**

These results indicate that the contrasting firmness of ‘2017D’ and ‘Duke’ is associated with differences in cell wall remodeling and ripening-related transcriptional processes and support a role for VcβGAL369 in promoting blueberry fruit softening.

## 1 Introduction

Blueberry (*Vaccinium*) is recognized for its significant health benefits, which are largely attributed to the flavonoids, mineral elements, and anthocyanins in the fruits (Tian et al., 2024). The postharvest physiology of blueberry is characterized by a rapid reduction in fruit firmness, with significant loss of structural integrity often occurring within days following harvest under non-refrigerated conditions. This physiological decline significantly curtails the marketable shelf life and impairs key organoleptic qualities, including firmness, juiciness, and flavor. Firmness at harvest has been associated with subsequent softening (Moggia et al., 2017) and is therefore important for storability, transportability, and commercial value (Chowdhury et al., 2023; Shi et al., 2023a). Consequently, a comprehensive understanding of the physiological, biochemical, and molecular mechanisms involved in blueberry ripening and softening is essential. Integrating genomics with postharvest physiology may provide a scientific basis for developing genetic strategies to improve firmness retention and extend shelf life (Ferrão et al., 2024).

Fruit ripening is a coordinated physiological process involving dynamic changes across various cellular compartments, including the cell wall, plastids, vacuole, and nucleus (Li et al., 2019). These changes are regulated by hormonal signaling, genetic programs, developmental cues, and environmental factors (Li et al., 2021). In addition to changes in internal tissues, cuticular properties have been associated with postharvest firmness in fleshy fruits, including blueberry (Moggia et al., 2016; Lara et al., 2019). Within this broader context, cell wall remodeling remains an important contributor to fruit softening during ripening and postharvest storage (Chea et al., 2019).

Cell wall remodeling involves enzymes and proteins involved in cell wall modification, each with distinct functions. These include pectin methylesterase (PME), polygalacturonase (PG), pectate lyase (PL), endo-1,4-β-glucanase (CEL/Cx), β-galactosidase (β-GAL), expansins (EXPs), and xyloglucan endotransglucosylase/hydrolases (XTHs) (Shi et al., 2023b). These proteins act on different wall components and influence cell-to-cell adhesion and tissue texture through distinct mechanisms. For example, overexpression of an XTH gene in tomato reduced xyloglucan depolymerization and fruit softening (Miedes et al., 2010). In another study, repression of the transcription factor *SlLOB1* delayed tomato fruit softening by reducing the expression of multiple cell wall-related genes (Shi et al., 2021). Transcriptomic analysis in kiwifruit also identified ripening-associated changes in genes involved in cell wall modification and hormone signaling (Chen et al., 2021).

Among these proteins, β-GAL is of particular interest because it removes terminal galactosyl residues from pectic side chains and has been linked to changes in fruit texture (Lazan et al., 2004; Yang et al., 2018). Functional studies further support its involvement in fruit softening. Down-regulation of *TBG4* reduced fruit softening in tomato (Smith et al., 2002), whereas heterologous overexpression of the persimmon β-galactosidase gene *DkGAL1* in tomato accelerated fruit ripening and reduced fruit firmness (Ban et al., 2018). However, whether specific β-GAL family members contribute to firmness variation in blueberry remains unclear.

In this study, we compared soft-fruited cultivar ‘Duke’ with the firm-fruited genotype ‘2017D’ to examine changes in fruit firmness, cell wall polysaccharide composition, and key enzyme activities during development and ripening. We also examined mesocarp tissue structure to relate these biochemical changes to differences in cellular organization. RNA-seq was performed on blue-stage fruit to identify genotype-associated expression differences potentially related to cell wall remodeling and firmness variation. We then examined the expression and function of *VcβGAL369* through developmental expression profiling, transient gene silencing, and overexpression assays. Together, these analyses provide insight into how cell wall metabolism is associated with the contrasting firmness of these two blueberry genotypes and may provide useful candidate genes for future studies aimed at improving fruit firmness and postharvest quality.

## 2 Materials and methods

### 2.1 Plant materials and sampling

The commercial blueberry cultivar ‘Duke’ and the firm-fruited genotype ‘2017D’ were used in this study. The genetic identity of ‘2017D’ was assessed using simple sequence repeat (SSR) markers (**Supplementary Figure S1**). All plant materials were grown at the Small Berry Genetic Breeding Center of Jilin Agricultural University, Changchun, China (43°48’N, 125°25’E; 259 m above sea level). Plants were maintained under the same field management conditions. The soil was sulfur-amended sandy loam supplemented with commercial peat, with an organic matter content of 3.72% and a pH of 5.5-5.8.

For each genotype, three individual plants were used as independent biological replicates. Fruits were sampled at four developmental stages defined according to fruit color and days post-anthesis (dpa): green (G, 35 dpa), turning (H, 50 dpa), purple (P, 60 dpa), and blue (B, 75 dpa). At each developmental stage, uniformly sized and disease-free fruits were randomly collected from different positions on each plant. Samples from the three plants were kept separate throughout collection and subsequent processing to preserve plant-level biological replication. Where pooling was required for biochemical or molecular analyses, fruits or fruit tissues were pooled within each plant rather than across plants. Samples designated for biochemical and molecular analyses were immediately frozen in liquid nitrogen and stored at −80 °C until use.

### 2.2 Evaluation of fruit texture and physicochemical traits

Fruit texture and physicochemical traits were evaluated using freshly harvested fruits. For each genotype and developmental stage, fruits collected from the three biological replicate plants were maintained separately according to plant origin. Fruit texture was evaluated by texture profile analysis (TPA) using a texture analyzer (TMS-PRO, Food Technology Corporation, USA) fitted with a P/75 probe (75 mm diameter). Two intact fruits were randomly selected from each plant and measured individually, resulting in six fruit measurements for each genotype × developmental-stage combination. The mean of the two fruits from each plant was used as the value for that biological replicate. Each fruit was compressed to 20% of its height. The test parameters were set as follows: pre-test speed of 5 mm/s, test speed of 2 mm/s, and post-test speed of 100 mm/min, 20% compression strain, a 2 s interval between two compression cycles, and a trigger force of 0.2 N. The texture parameters, including firmness, springiness, chewiness, gumminess, resilience, and cohesiveness, were derived from the TPA curves (Wang et al., 2024). Firmness values were converted to Newtons (N) using the conversion factor of 1 N ≈ 102 gf for standardized reporting. SSC and TA were measured separately for fruits from each plant using a portable refractometer and acidimeter (PA2-BX1ACID7) calibrated for blueberry fruit. SSC was expressed as °Brix, and TA was expressed as a percentage.

### 2.3 Determination of cell wall components

For each genotype and developmental stage, frozen fruit tissues from the three sampled plants were processed separately for biochemical analyses, with each plant constituting one biological replicate. Water-soluble pectin (WSP) and insoluble pectin contents were quantified according to a previously described method (Liu et al., 2023) with appropriate modifications. Briefly, 1.0 g of frozen fruit powder was homogenized and extracted with 25 mL of 95% ethanol at 95 °C for 30 min. After cooling to room temperature, the mixture was centrifuged at 8,000 × g for 15 min. This ethanol washing procedure was repeated three times until the supernatant became colorless. The resulting precipitate, representing the alcohol-insoluble solids (AIS), was resuspended in 20 mL of distilled water and incubated at 50 °C for 30 min to extract water-soluble pectin (WSP). After subsequent cooling, the solution was centrifuged at 9,000 × g for 15 minutes to isolate the supernatant containing WSP. The remaining pellet was subsequently used for insoluble pectin extraction following the same procedure with 25.0 mL of 0.5 mol L^-1^ sulfuric acid solution. Pectin contents were determined colorimetrically using galacturonic acid as the standard.

In accordance with the manufacturer’s guidelines, the contents of cellulose and hemicellulose were evaluated with the Hemicellulose Content Assay Kit and Cellulose Content Assay Kit (BOX BIO, Beijing, China).

### 2.4 Cell wall degrading enzymes

Cellulase activity (EC 3.2.1.4) (Njie et al., 2023) was assayed by incubating 0.5 mL of supernatant with 1.5 mL of 1% (w/v) carboxymethyl cellulose (CMC) in a 37 °C water bath for 1 h. The released reducing sugars were measured by adding 1.5 mL of DNS reagent, heating for 5 minutes in a boiling water bath, cooling, and diluting the mixture to 25 mL. Absorbance was measured at 540 nm. Cellulase activity was expressed as U kg^-1^ FW.

PG activity (EC 3.2.1.15) (Ye et al., 2024) was measured in a 1 h incubation at 37 °C in a reaction mixture containing 1.0 mL of sodium acetate buffer (50 mmol L^-1^, pH 5.5), 0.5 mL of 1% (w/v) polygalacturonic acid, and 0.5 mL of crude enzyme. The reaction was terminated with 1.5 mL of DNS, heated for 5 minutes, then cooled and diluted to 25 mL before measuring the absorbance at 540 nm. PG activity was expressed as U kg^-1^ FW.

β-GAL activity was measured using a kit (BOX BIO, Beijing, China) following the manufacturer’s instructions. β-GAL activity was expressed as U g^−1^.

Pectin methylesterase (PME, EC 3.1.1.11) activity was measured using the bromothymol blue colorimetric method with modifications (Hagerman and Austin, 1986). Frozen fruit powder was homogenized in extraction solution containing 8.8% (w/v) NaCl. The homogenate was incubated on ice and centrifuged at 12,000 × g for 20 min at 4 °C. The reaction mixture contained 0.5% citrus pectin, 0.01% bromothymol blue, and crude enzyme extract. Absorbance was recorded at 620 nm after reaction at room temperature. PME activity was calculated using a calibration curve prepared under the same reaction conditions and expressed as U kg^-1^ FW.

### 2.5 Paraffin sectioning and histological observation

Mesocarp cell morphology was quantified from calibrated paraffin-section images obtained at 35 and 75 dpa. For each genotype and developmental stage, three independent fruits were randomly selected for morphometric analysis. Following a general histological framework previously applied to blueberry fruit (Wan et al., 2024), a standardized sampling region extending 400 μm along the peel and 0–200 μm inward from the peel was analyzed using Fiji. Closed mesocarp cell profiles were detected semi-automatically and manually verified. Profiles were retained when the cell-profile area ranged from 150 to 8,000 μm², solidity was ≥ 0.70, circularity was ≥ 0.10, aspect ratio was ≤ 6, and the centroid was located within the predefined sampling region. Cell-profile area and the dimensions parallel (periclinal) and perpendicular (anticlinal) to the peel surface were recorded. For each fruit, the mean value of the retained cell profiles was calculated and used for subsequent statistical analysis.

### 2.6 RNA extraction and library construction

Fruits at the blue stage (75 dpa) of ‘Duke’ and ‘2017D’ were used for RNA-seq analysis. For each genotype, three individual plants served as independent biological replicates. Each biological replicate consisted of three whole fruits collected from a single plant, and samples from different plants were processed separately. Total RNA was extracted from whole-fruit tissue using a modified CTAB method. RNA purity and concentration were assessed using a NanoDrop spectrophotometer, and RNA integrity was evaluated using an Agilent 2100 Bioanalyzer.

RNA-seq library construction and sequencing were performed by BGI Life Tech Co., Ltd. (Wuhan, China). mRNA was enriched using oligo(dT)-conjugated magnetic beads and fragmented into short fragments. First-strand cDNA was synthesized using random hexamer primers, followed by second-strand cDNA synthesis. After end repair, A-tailing, and adapter ligation, the cDNA fragments were amplified by PCR and purified using AMPure XP beads. Library quality was assessed using an Agilent 2100 Bioanalyzer. The final libraries were converted into DNA nanoballs and sequenced on the DNBSEQ-G400 platform in paired-end mode, generating 100-bp reads from each end (2 × 100 bp). The RNA-seq data generated in this study have been deposited in the NCBI Sequence Read Archive under BioProject accession number PRJNA1481787.

### 2.7 RNA-seq data processing and differential expression analysis

Raw sequencing reads were filtered using SOAPnuke v1.4.0 to remove adapter-containing reads, reads with more than 5% unknown bases, and low-quality reads. Clean reads were aligned to the Vaccinium corymbosum cv. Draper v1.0 reference genome using Subjunc implemented in Rsubread v2.16.1. Only uniquely mapped read pairs were retained for subsequent gene-level quantification. Fragment counts were generated using featureCounts based on annotated exons under a paired-end, unstranded counting scheme.

Differential expression between ‘2017D’ and ‘Duke’ was analyzed using DESeq2 v1.42.1 with the unnormalized integer fragment-count matrix as input. Genes with fewer than 10 fragments across all six libraries were excluded prior to differential expression analysis. P values were adjusted for multiple testing using the Benjamini–Hochberg procedure, and genes with an adjusted P value < 0.05 and |log_2_ fold change| ≥ 1 were considered differentially expressed. Positive log_2_ fold-change values indicate higher expression in ‘2017D’, whereas negative values indicate higher expression in ‘Duke’.

Gene expression abundance was additionally expressed as transcripts per million (TPM) and fragments per kilobase of transcript per million mapped fragments (FPKM), calculated from the assigned fragment counts and annotated gene lengths. TPM and FPKM values were used for expression reporting. Heatmaps were generated using log_2_(TPM + 1) values followed by row-wise z-score normalization.

### 2.8 Gene functional annotation and enrichment analysis

Gene functional annotations were obtained from the project annotation files, including annotations from the NR, Gene Ontology (GO), and Kyoto Encyclopedia of Genes and Genomes (KEGG) databases. Gene identifiers were matched to the Vaccinium corymbosum cv. Draper v1.0 gene models using the corresponding gene-ID mapping table. Transcription-factor families were assigned according to the transcription-factor classification file provided by the sequencing service.

GO and KEGG overrepresentation analyses were performed for all differentially expressed genes (DEGs) and separately for genes showing higher expression in each genotype. For each database, genes included in the differential expression analysis and carrying the corresponding functional annotation were used as the background set. Duplicate gene–term assignments were removed before enrichment analysis. Terms associated with 5–3,000 background genes were tested using a one-sided hypergeometric test. *P* values were adjusted using the Benjamini–Hochberg procedure, and terms with an FDR < 0.05 were considered significantly enriched. Significantly enriched GO terms and KEGG pathways associated with fruit ripening, hormone signaling, carbohydrate metabolism, and cell wall remodeling were further examined for candidate-gene screening.

### 2.9 Real-time quantitative PCR verification

For RNA-seq validation, the same RNA samples used for transcriptome sequencing were analyzed. For developmental expression profiling, RNA was extracted from fruit samples collected at the green, turning, purple and blue stages. First-strand cDNA was synthesized from total RNA using the PrimeScript™ RT Reagent Kit with gDNA Eraser (TaKaRa) according to the manufacturer’s protocol. Gene-specific primers were developed with the PrimerExpress software (**Supplementary Table S1**). The PCR amplification was operated using TB Green™ Premix Ex Taq™ II (Tli RNaseH Plus) (TaKaRa), with the *EF1α* (Deng et al., 2020) serving as the housekeeping gene. Relative expression levels were calculated using the 2^-ΔΔCt^ method. Each sample included three biological replicates and three technical replicates.

### 2.10 Identification and characterization of the VcBGAL gene family

Genome sequences and annotation files of Vaccinium corymbosum cv. Draper v1.0 were obtained from the Genome Database for Vaccinium (GDV). Candidate VcBGAL proteins were identified by BLASTP against the Draper v1.0 protein dataset in GDV using known plant GH35 β-galactosidases as queries (E-value ≤ 1 × 10^-5^) and by an HMM-based search using the Simple HMM Search function in TBtools-II v2.027 with the Pfam Glyco_hydro_35 profile PF01301.23 (E-value ≤ 1 × 10^-5^). Candidate sequences identified by the two approaches were combined, duplicate hits were removed, and conserved-domain composition was examined using NCBI Batch CD-Search. Proteins with conserved-domain support consistent with the GH35 β-galactosidase family were retained, resulting in 59 VcBGAL proteins for subsequent analyses.

VcBGAL protein sequences were aligned using MUSCLE v5.1 (-align -consiters 2 -refineiters 100) and trimmed using trimAl v1.4.rev15 with the automated1 algorithm. A maximum-likelihood phylogenetic tree was constructed using IQ-TREE v1.6.12. The WAG+I+G4 substitution model was selected by ModelFinder according to the Bayesian information criterion, and branch support was assessed using 1,000 ultrafast bootstrap replicates with NNI optimization (-bb 1000 -bnni).

Conserved motifs were identified using MEME v5.5.5 under the ZOOPS model, with a maximum of 10 motifs and motif widths ranging from 6 to 50 amino acids. Exon–intron structures were extracted from the Draper v1.0 GFF annotation file. The phylogenetic tree, motif distribution, conserved-domain composition, and gene structures were visualized using TBtools-II v2.097.

### 2.11 Virus-induced gene silencing in blueberry fruits

Virus-induced gene silencing (VIGS) of the *VcβGAL369* gene was performed in blueberry fruits using a tobacco rattle virus (TRV)-based system according to a previously established method (Han et al., 2021) with modifications. A 395-bp fragment corresponding to positions 7–401 of the VcβGAL369 coding sequence was selected for silencing. Fragment specificity was evaluated by BLASTn against the blueberry genome in the Genome Database for Vaccinium (GDV) and by sequence comparison with other VcBGAL family members using TBtools. The fragment was amplified using primers containing homology arms corresponding to the EcoRI and BamHI sites of pTRV2 and inserted into the vector by homologous recombination to generate pTRV2-VcβGAL369. The TRV system consisted of pTRV1, carrying the TRV RNA1 component required for viral replication and movement, and pTRV2, carrying either the VcβGAL369 fragment or no insert as the control.

The pTRV1, empty pTRV2, and pTRV2-VcβGAL369 constructs were separately introduced into Agrobacterium tumefaciens GV3103 (pSoup-19). Bacterial cultures were grown in LB medium supplemented with 50 μg mL^-1^ kanamycin and 50 μg mL^-1^ rifampicin at 28 °C until the OD_600_ reached 1.2. Cells were then harvested by centrifugation and resuspended in MMA infiltration buffer containing 10 mmol L^-1^ MES, 10 mmol L^-1^ MgCl_2_, and 200 μmol L^-1^ acetosyringone. Agrobacterium suspensions containing pTRV1 and either empty pTRV2 or pTRV2-VcβGAL369 were mixed at a 1:1 (v/v) ratio and incubated for 2 h at room temperature in the dark before infiltration.

Uniformly sized purple-stage ‘Duke’ fruits were infiltrated with the corresponding Agrobacterium suspensions using a 1 mL sterile syringe. Fruits infiltrated with pTRV1 plus empty pTRV2 were designated as TRV:00, whereas those infiltrated with pTRV1 plus pTRV2-VcβGAL369 were designated as TRV: VcβGAL369. Treated fruits were labeled, covered with breathable bags, and maintained on the plants under field conditions. Fruits were harvested 14 days after infiltration for phenotypic observation, qRT-PCR, firmness measurement, cell wall component analysis, and enzyme activity assays. Each treatment comprised three independent biological replicates, with 30 fruits per replicate.

### 2.12 Transient overexpression of VcβGAL369 in blueberry fruit

The coding sequence (CDS) of *VcβGAL369* was cloned and inserted into the overexpression vector pGreenII62-SK to create the pGreenII62-SK-VcβGAL369 construct. This construct was subsequently transformed into *Agrobacterium tumefaciens* strain EHA105 for transient overexpression assays. For functional assessment, bacterial suspensions harboring either the *VcβGAL369* overexpression construct or the empty vector were injected into uniformly sized purple-stage fruits using a syringe. The samples were harvested and photographed approximately 3 days later. Because TRV-mediated gene silencing requires a longer period to become established than Agrobacterium-mediated transient overexpression, fruits were harvested 14 days after infiltration for VIGS and 3 days after infiltration for transient overexpression. Each treatment comprised three independent biological replicates, with 30 fruits per replicate.

### 2.13 Statistical analysis

Data are presented as mean ± standard deviation (SD). Statistical analyses were performed using SPSS 23.0 and R 4.3.2. Fruit texture and physicochemical traits, cell wall components, and related enzyme activities were analyzed using two-way analysis of variance (ANOVA), with genotype and developmental stage as fixed factors and their interaction included in the model. Multiple comparisons among genotype × developmental-stage combinations were performed using Tukey’s honestly significant difference (HSD) test. Histomorphometric data were analyzed using linear models including genotype, developmental stage, their interaction, and imaging batch, and within-stage genotype contrasts were adjusted using the Holm method. Comparisons between control and treatment groups in the VIGS and transient overexpression experiments were performed using two-tailed Student’s t-test. A P value < 0.05 was considered statistically significant.

## 3 Results

### 3.1 Phenotypic, textural, and physicochemical characterization of blueberry fruit

Comparative analysis of ‘Duke’ and the firm-fruited genotype ‘2017D’ revealed pronounced differences in fruit texture and physicochemical traits during fruit development (**Figure 1**). Both genotypes showed a similar progression in external coloration from the green to blue stages (**Figure 1A**), whereas their firmness profiles differed markedly. Firmness in ‘Duke’ declined from 21.27 N at the green stage to 3.32 N at the blue stage, while ‘2017D’ maintained substantially higher firmness, decreasing from 28.77 N to 17.81 N over the same developmental period (**Figure 1B**).

**Figure 1:**
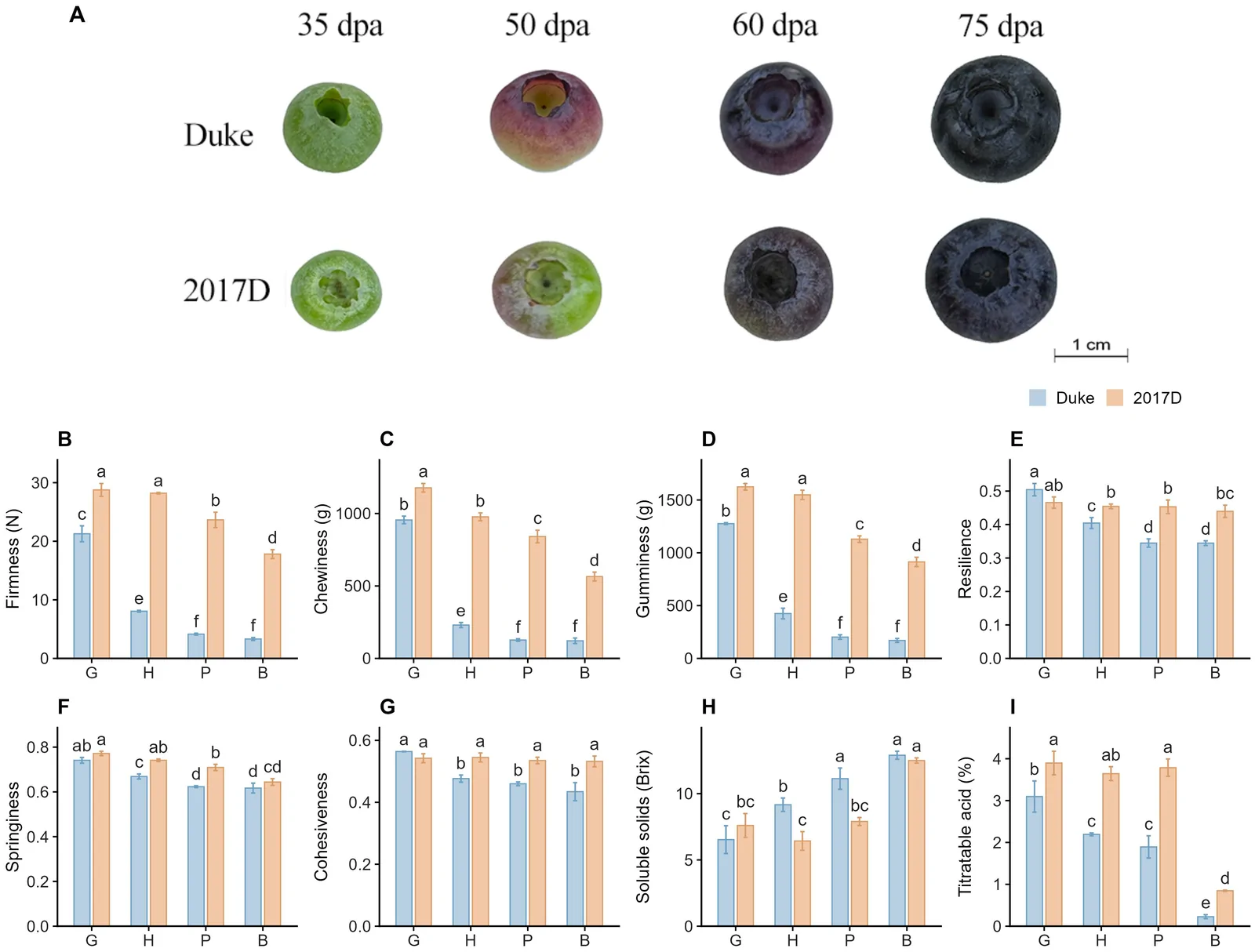
Phenotypic, textural, and physicochemical characteristics of ‘Duke’ and the firm-fruited blueberry genotype ‘2017D’ during fruit development. **(A)** Representative fruits of ‘Duke’ and ‘2017D’ at 35, 50, 60 and 75 days post-anthesis (dpa), corresponding to the green **(G)**, turning **(H)**, purple **(P)** and blue **(B)** stages, respectively. Scale bar = 1 cm. **(B)** Firmness. **(C)** Chewiness. **(D)** Gumminess. **(E)** Resilience. **(F)** Springiness. **(G)** Cohesiveness. **(H)** Soluble solids. **(I)** Titratable acid. Data are presented as mean ± SD (n = 3). Different lowercase letters indicate significant differences according to Tukey’s HSD test (*P* < 0.05). G, green stage; H, turning stage; P, purple stage; B, blue stage.

Texture profile analysis further revealed contrasting developmental patterns between the two genotypes. Chewiness and gumminess remained significantly higher in ‘2017D’ than in ‘Duke’ throughout development, with particularly pronounced differences from the turning stage onward (**Figures 1C, D**). Resilience declined substantially in ‘Duke’ after the green stage, whereas ‘2017D’ maintained significantly higher resilience from the turning stage onward (**Figure 1E**). Springiness did not differ significantly between the two genotypes at the green or blue stages but was significantly higher in ‘2017D’ at the turning and purple stages (**Figure 1F**). Cohesiveness decreased after the green stage in ‘Duke’ but remained comparatively stable in ‘2017D’, resulting in significantly higher cohesiveness in ‘2017D’ from the turning stage onward (**Figure 1G**).

Soluble solids generally increased toward the blue stage in both genotypes, although their developmental patterns differed (**Figure 1H**). Titratable acidity declined during development in both genotypes and remained significantly higher in ‘2017D’ than in ‘Duke’ at each developmental stage (**Figure 1I**). Overall, ‘2017D’ exhibited slower firmness loss and greater retention of several texture attributes during fruit development.

### 3.2 Histological comparison of mesocarp structure between ‘2017D’ and ‘Duke’ during fruit ripening

Paraffin sections were used to compare mesocarp tissue structures between ‘Duke’ and ‘2017D’ at the green and blue stages (**Figure 2**). At the green stage, both genotypes exhibited relatively well-organized mesocarp tissues, with no significant differences in cell-profile area or cell dimensions (**Supplementary Figure S2**). At the blue stage, tissue disorganization was more evident in ‘Duke’, with a less regular cellular arrangement and greater disruption of tissue continuity. In contrast, ‘2017D’ retained a comparatively more continuous mesocarp organization. Morphometric analysis further showed that ‘2017D’ had a significantly larger cell-profile area and greater periclinal and anticlinal diameters than ‘Duke’ at 75 dpa (**Supplementary Figure S2**).

**Figure 2:**
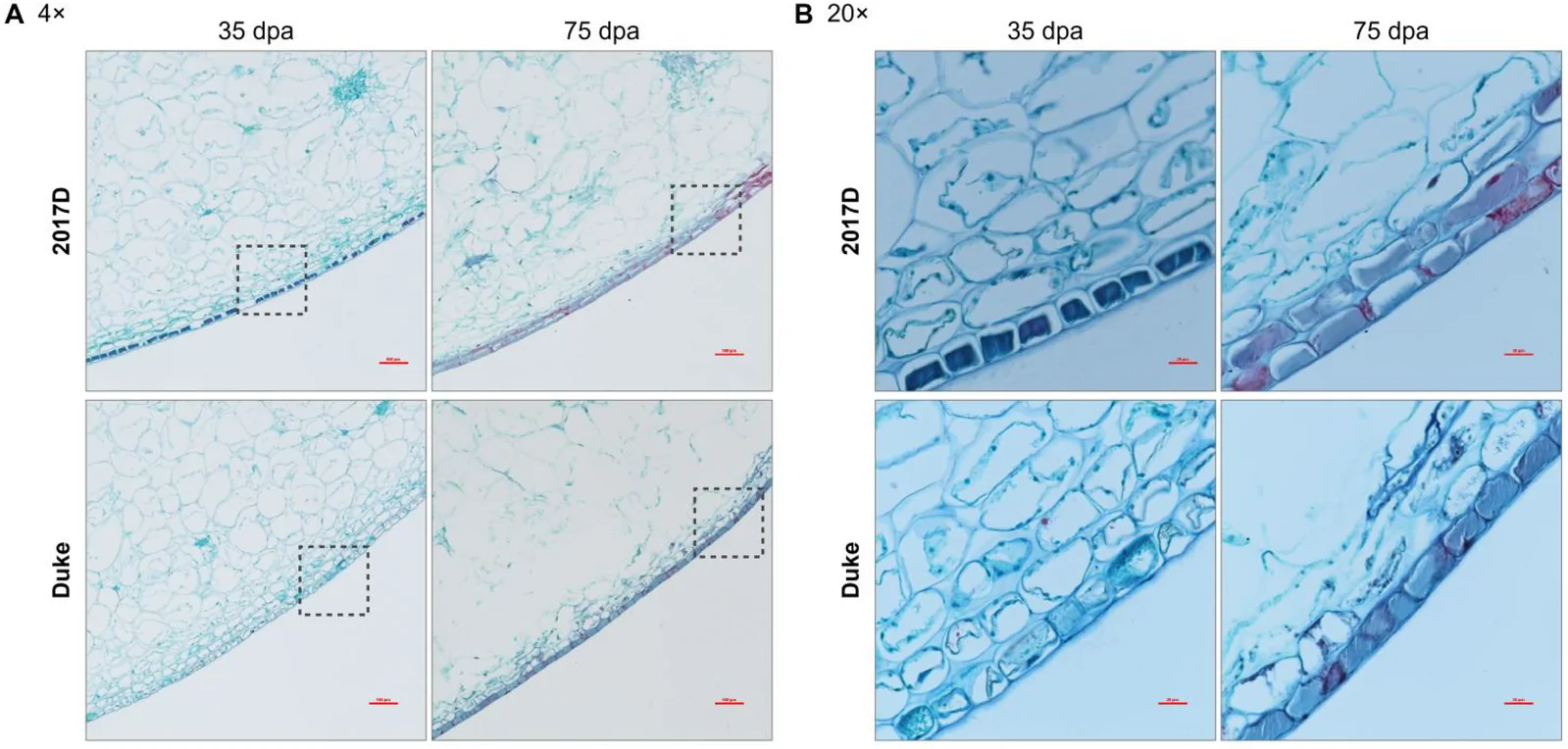
Histological comparison of mesocarp structure between ‘2017D’ and ‘Duke’ at the green and blue stages. **(A)** Representative low-magnification paraffin sections (4×) at 35 and 75 days post-anthesis (dpa). Dashed boxes indicate the regions shown at higher magnification in **(B)**. **(B)** Higher-magnification views (20×) of the corresponding mesocarp regions. Scale bars = 100 µm in **(A)** and 20 µm in **(B)**.

### 3.3 Changes in cell wall components and related enzyme activities during fruit development

Distinct changes in cell wall composition and related enzyme activities were observed between ‘Duke’ and the firm-fruited genotype ‘2017D’ during fruit development (**Figure 3**). Compared with ‘Duke’, ‘2017D’ generally maintained higher levels of structural polysaccharides, with significantly higher cellulose contents at the green, turning, and purple stages and higher hemicellulose contents throughout development (**Figures 3A, B**). Pectin fractions also showed distinct developmental patterns between the two genotypes. ‘2017D’ maintained significantly higher insoluble pectin contents across all developmental stages, whereas ‘Duke’ accumulated significantly more soluble pectin at the purple and blue stages (**Figures 3C, D**).

**Figure 3:**
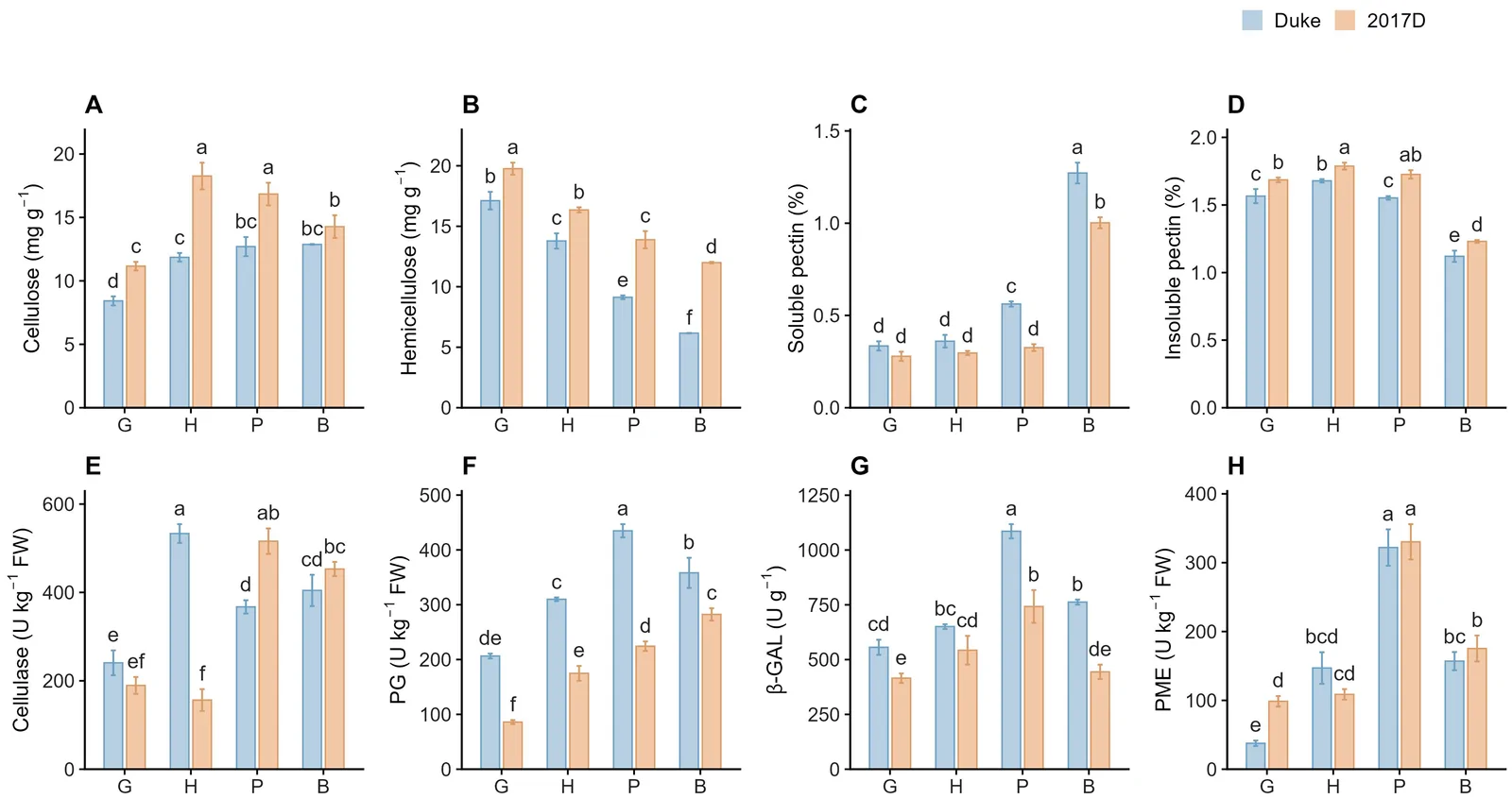
Dynamics of cell wall components and related enzyme activities during blueberry fruit development. **(A)** Cellulose content. **(B)** Hemicellulose content. **(C)** Soluble pectin content. **(D)** Insoluble pectin content. **(E)** Cellulase activity. **(F)** PG activity. **(G)** β-GAL activity. **(H)** PME activity. Data are presented as mean ± SD (n = 3). Different lowercase letters indicate significant differences according to Tukey’s HSD test (*P* < 0.05). G, green stage; H, turning stage; P, purple stage; B, blue stage.

The two genotypes also differed in cell wall-related enzyme activities. Cellulase activity was significantly lower in ‘2017D’ at the turning stage but significantly higher at the purple stage, whereas no significant differences were detected at the green or blue stages (**Figure 3E**). In contrast, PG activity was consistently lower in ‘2017D’ than in ‘Duke’ throughout development (**Figure 3F**). β-GAL activity was lower in ‘2017D’ at the green, purple, and blue stages, whereas no significant difference was detected at the turning stage (**Figure 3G**). PME activity varied across developmental stages, with a significant difference between the two genotypes detected only at the green stage (**Figure 3H**). Together, these results indicate that the firm-fruited phenotype of ‘2017D’ was associated with greater retention of structural polysaccharides and insoluble pectin, together with lower PG and β-GAL activities during ripening.

### 3.4 RNA-seq profiling and differential expression analysis at the blue fruit stage

Because the blue stage represents the fully ripe stage and a marked difference in fruit firmness remained between ‘Duke’ and ‘2017D’ at this stage, blue-stage fruits were selected for transcriptome analysis. Six RNA-seq libraries were constructed, with three independent biological replicates for each genotype. After quality filtering, a total of 394.37 million clean reads were obtained, with 63.64–70.48 million reads per library. The proportion of read pairs uniquely mapped to the Vaccinium corymbosum cv. Draper v1.0 reference genome ranged from 57.60% to 60.14%, and 84.05–85.88% of the uniquely mapped fragments were assigned to annotated gene exons (**Supplementary Table S2**).

Principal component analysis (PCA) based on variance-stabilized gene counts clearly separated ‘2017D’ and ‘Duke’ along PC1, which explained 88.3% of the total variance, while PC2 accounted for an additional 3.7% (**Figure 4A**). Differential expression analysis identified 6,168 differentially expressed genes (DEGs) between the two genotypes using an adjusted P value < 0.05 and |log_2_ fold change| ≥ 1 as thresholds. Among these, 3,835 genes showed higher expression in ‘2017D’, whereas 2,333 showed higher expression in ‘Duke’ (**Figure 4B**).

**Figure 4:**
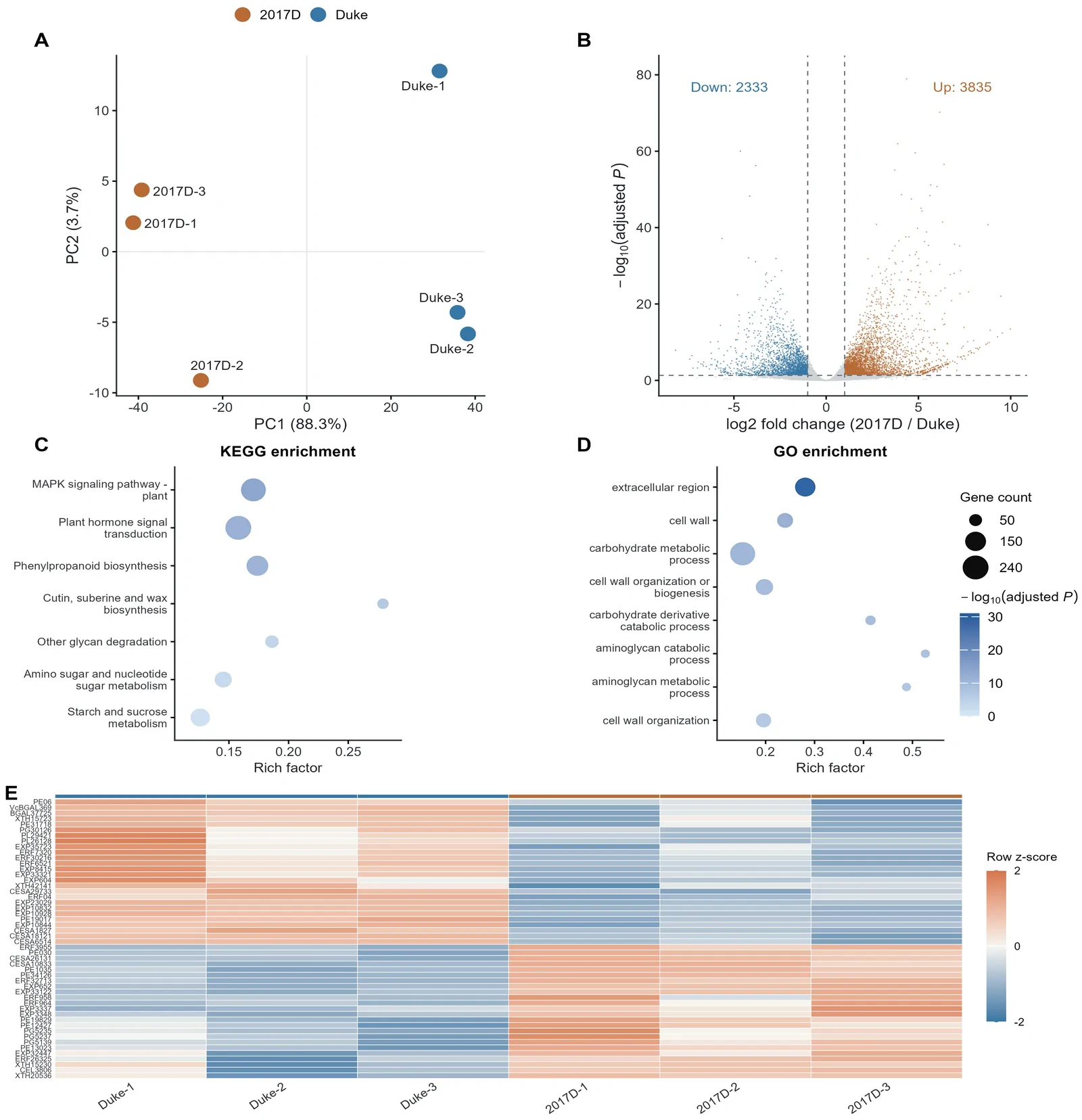
Transcriptomic overview and pathway-guided screening of cell wall-related candidate genes in ‘2017D’ and ‘Duke’ at the blue fruit stage. **(A)** Principal component analysis (PCA) of six RNA-seq libraries based on variance-stabilized gene counts. **(B)** Volcano plot of differentially expressed genes (DEGs) between ‘2017D’ and ‘Duke’. Orange indicates genes expressed at higher levels in ‘2017D’, blue indicates genes expressed at higher levels in ‘Duke’, and gray indicates genes that did not meet the differential-expression criteria. Dashed lines indicate an adjusted P value < 0.05 and |log_2_ fold change| ≥ 1. **(C)** Selected significantly enriched KEGG pathways among the DEGs. Bubble size indicates DEG number, and color indicates −log_10_(adjusted P). **(D)** Selected significantly enriched GO terms among the DEGs. Bubble size indicates DEG number, and color indicates −log_10_(adjusted P). **(E)** Heatmap of 50 cell wall-related candidate genes selected based on functional enrichment, gene annotation, and reported roles in fruit softening. Expression values are shown as row Z-scores calculated from log_2_(TPM + 1). Rows were clustered by expression pattern, whereas columns were arranged by genotype.

### 3.5 Functional enrichment analysis highlights pathways related to ripening and cell wall metabolism

To identify biological processes associated with the transcriptional differences between ‘2017D’ and ‘Duke’, KEGG and GO enrichment analyses were performed on the DEGs. Several significantly enriched KEGG pathways were related to hormone signaling, carbohydrate metabolism, and cell wall-associated processes (**Figure 4C**), including MAPK signaling pathway–plant, plant hormone signal transduction, phenylpropanoid biosynthesis, starch and sucrose metabolism, amino sugar and nucleotide sugar metabolism, other glycan degradation, and cutin, suberine and wax biosynthesis.

GO enrichment analysis further identified terms associated with the extracellular region, cell wall, carbohydrate metabolic process, cell wall organization or biogenesis, carbohydrate derivative catabolic process, aminoglycan catabolic process, aminoglycan metabolic process, and cell wall organization (**Figure 4D**). These enrichment patterns indicated that the transcriptional differences between the two genotypes involved multiple processes related to carbohydrate metabolism and cell wall organization, consistent with their contrasting fruit texture at the blue stage.

### 3.6 Screening and expression profiling of cell wall-related candidate genes

Based on the KEGG and GO enrichment results, together with gene functional annotations and thereported roles of these gene families in fruit softening, 50 differentially expressed genes associated with cell wall remodeling were initially screened for further expression profiling (**Supplementary Table S3**). These genes included expansins (EXPs), xyloglucan endotransglucosylase/hydrolases (XTHs), pectin esterases (PEs), cellulose synthases and cellulose synthase-like proteins, polygalacturonases (PGs), pectate lyases (PLs), β-galactosidases (β-GALs), cellulases (CELs), and ethylene-responsive transcription factors (ERFs). Hierarchical clustering divided the 50 candidate genes into two major expression groups, with relatively higher expression in ‘2017D’ or ‘Duke’, respectively (**Figure 4E**). Among these genes, 24 showed higher expression in ‘2017D’ and 26 showed higher expression in ‘Duke’. The β-GAL and PL candidates were consistently expressed at higher levels in ‘Duke’, whereas EXP, XTH, PE, PG, CesA, and ERF family members were represented in both expression groups. These contrasting expression patterns were further examined by qRT-PCR using representative candidate genes.

### 3.7 Expression profiling of candidate genes and structural characterization of VcβGAL369

To validate the transcriptomic expression patterns, 16 representative candidate genes were examined by qRT-PCR at the blue fruit stage (**Figure 5A**). The qRT-PCR results were generally consistent in direction with the RNA-seq data. Developmental expression profiling of 11 selected genes further revealed distinct genotype- and stage-dependent patterns (**Figure 5B**). During the later developmental stages, *VcEXP35921* and *VcEXP33122* showed relatively higher expression in ‘2017D’, whereas *VcβGAL369* and *VcPG30126* showed higher expression in ‘Duke’ during ripening.

**Figure 5:**
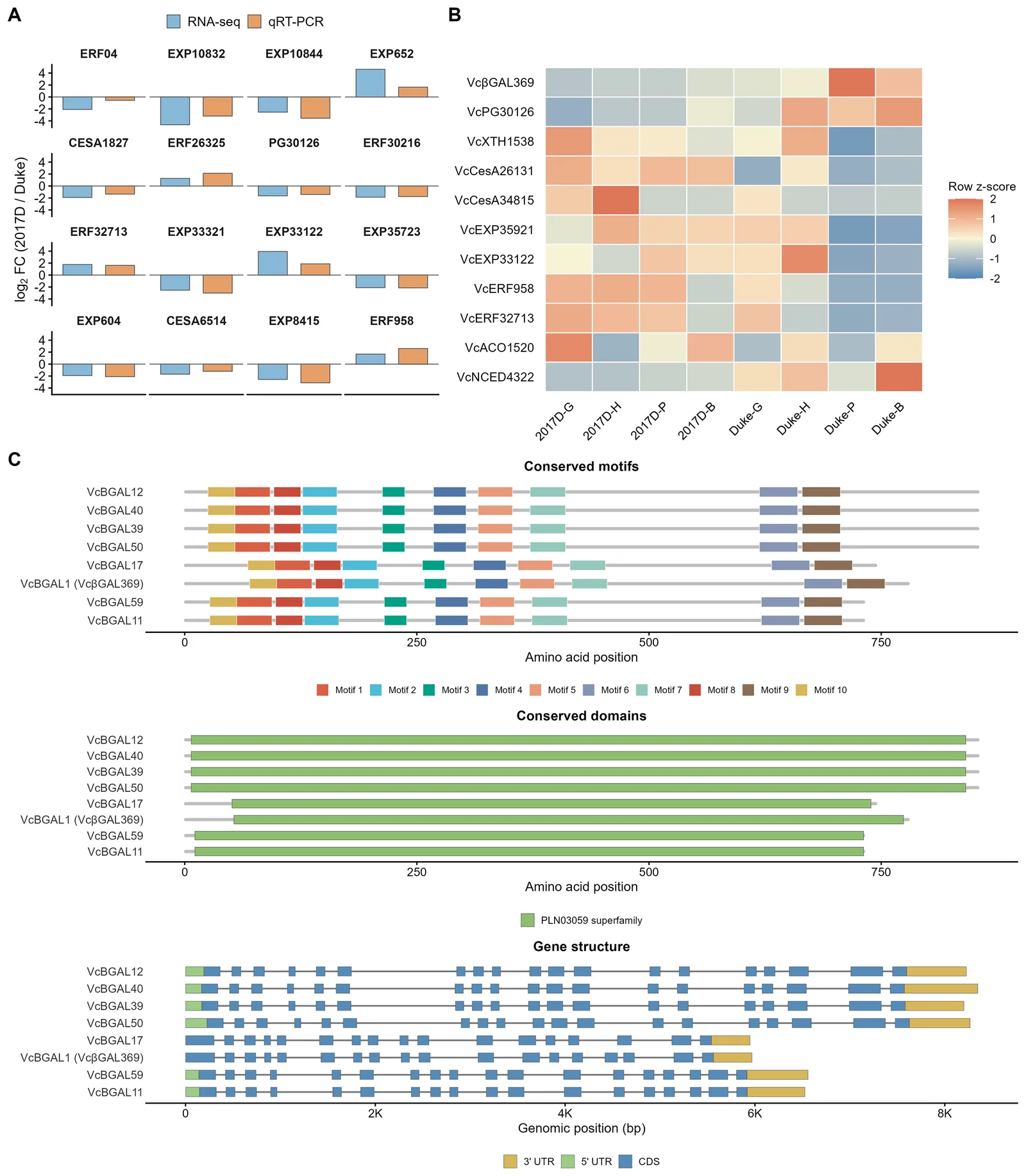
Expression profiling of candidate genes and structural characterization of VcBGAL family members in ‘Duke’ and ‘2017D’ blueberry. **(A)** Comparison of RNA-seq and qRT-PCR log_2_ fold-change values for 16 selected candidate genes in blue-stage fruit. Values are expressed as log_2_FC (2017D/Duke). **(B)** Heatmap showing qRT-PCR expression patterns of 11 selected candidate genes during fruit development in ‘2017D’ and ‘Duke’. Expression values were log_2_-transformed and normalized by row Z-score. G, green stage; H, turning stage; P, purple stage; B, blue stage. **(C)** Conserved motif, conserved-domain, and gene-structure analyses of VcBGAL1 (*VcβGAL369*) and seven closely related VcBGAL family members. Different colored boxes represent conserved motifs. Conserved-domain matches were identified using NCBI CDD, and coding sequences (CDS), 5′ untranslated regions (5′ UTRs), 3′ untranslated regions (3′ UTRs), and introns are shown according to their annotated positions.

Among these genes, *VcβGAL369* showed a pronounced expression difference between the two genotypes in the RNA-seq analysis together with a distinct developmental expression pattern. Its higher expression in ‘Duke’ during ripening was also consistent with the higher β-GAL activity observed in the softer genotype at several developmental stages. These expression and biochemical characteristics, together with its annotation as a putative β-galactosidase involved in cell wall polysaccharide modification, supported the selection of *VcβGAL369* for further structural characterization. Analysis of 59 VcBGAL family members showed that *VcβGAL369* clustered with a group of closely related proteins sharing broadly similar motif composition, conserved-domain patterns, and gene structures (**Supplementary Figure S3**). Further comparison of *VcβGAL369* with seven closely related VcBGAL members showed similar arrangements of major conserved motifs and CDD matches (**Figure 5C**). Comparison with representative GH35 β-galactosidases further showed that *VcβGAL369* contained the conserved QIENE and WTEAW motifs, including the putative catalytic residues Glu183 and Glu252 (**Supplementary Figure S4**). Structural prediction of its catalytic region was consistent with these conserved sequence features. Together, these findings provided additional support for the subsequent functional analysis of *VcβGAL369.*

### 3.8 Transient silencing of VcβGAL369 in blueberry fruit

To assess the role of *VcβGAL369* in blueberry fruit softening, VIGS was performed using TRV:00 as the empty-vector control. At 14 days post-infiltration, *VcβGAL369* expression was significantly reduced in TRV: VcβGAL369 fruit, confirming effective silencing (**Figure 6C**). VcβGAL369-silenced fruit exhibited significantly higher firmness than TRV:00 fruit (**Figures 6A, B**). β-GAL activity was also significantly reduced in silenced fruit (**Figure 6D**).

**Figure 6:**
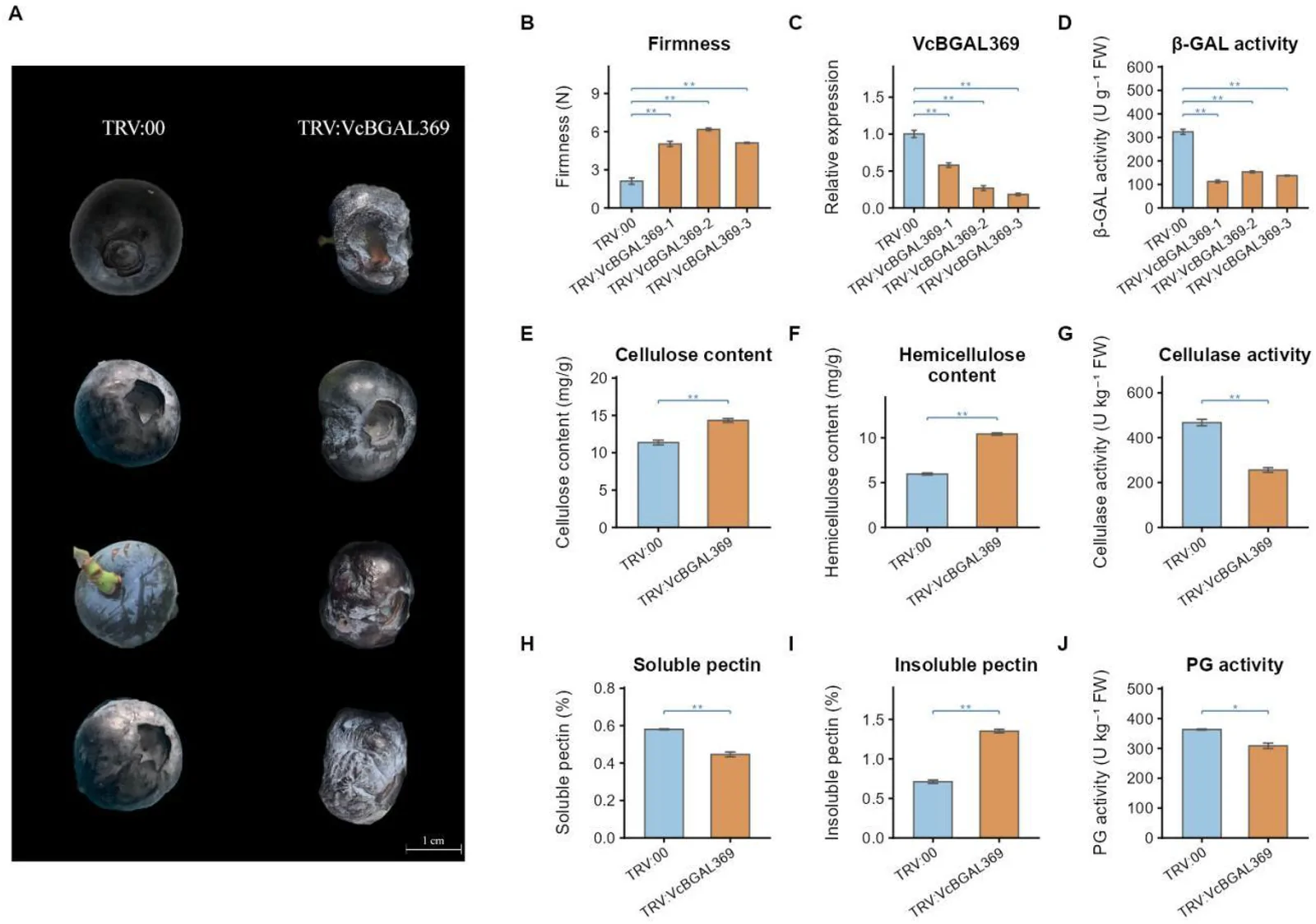
Effects of *VcβGAL369* silencing on blueberry fruit firmness and cell wall metabolism. **(A)** Representative phenotypes of TRV:00 and TRV: VcβGAL369 fruit at 14 days post-infiltration. Scale bar = 1 cm. **(B)** Fruit firmness. **(C)** Relative expression level of *VcβGAL369*. **(D)** β-GAL activity. **(E)** Cellulose content. **(F)** Hemicellulose content. **(G)** Cellulase activity. **(H)** Soluble pectin content. **(I)** Insoluble pectin content. **(J)** PG activity. Data are presented as means ± SD. Significance was determined using two-tailed Student’s t-test: *p < 0.05, **p < 0.01.

Cell wall analysis further revealed changes associated with *VcβGAL369* silencing. Cellulose and hemicellulose contents were significantly higher in silenced fruit than in TRV:00 fruit (**Figures 6E, F**). Soluble pectin content decreased, whereas insoluble pectin content increased (**Figures 6H, I**). The activities of cellulase and polygalacturonase were also reduced in silenced fruit (**Figures 6G, J**). These results indicate that transient silencing of *VcβGAL369* increased fruit firmness and promoted the retention of cell wall structural components.

### 3.9 Transient overexpression of VcβGAL369 in blueberry fruit

To further examine the function of *VcβGAL369*, transient overexpression was performed in blueberry fruit using the empty vector as the control. *VcβGAL369* transcript levels were significantly increased in VcβGAL369-OE fruit at 3 days post-infiltration (**Figure 7C**). β-GAL activity was also significantly higher in overexpressing fruit than in the EV control (**Figure 7D**).

**Figure 7:**
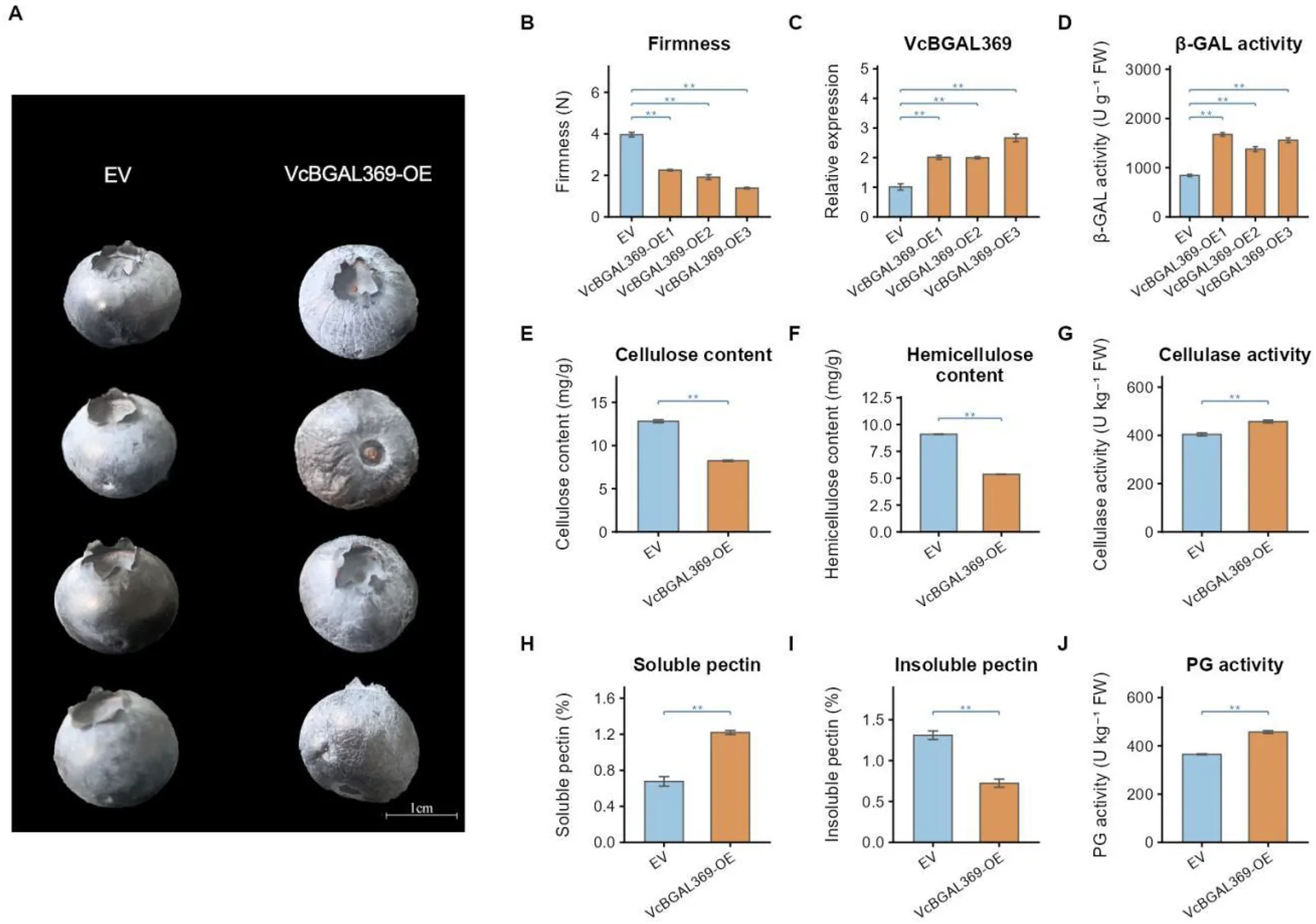
Effects of *VcβGAL369* overexpression on blueberry fruit firmness and cell wall metabolism. **(A)** Representative phenotypes of EV and VcβGAL369-OE fruit at 3 days post-infiltration. Scale bar = 1 cm. **(B)** Fruit firmness. **(C)** Relative expression level of *VcβGAL369*. **(D)** β-GAL activity. **(E)** Cellulose content. **(F)** Hemicellulose content. **(G)** Cellulase activity. **(H)** Soluble pectin content. **(I)** Insoluble pectin content. **(J)** PG activity. Data are presented as means ± SD. Significance was determined using two-tailed Student’s t-test: *p < 0.05, **p < 0.01.

Transient overexpression of *VcβGAL369* resulted in a clear softening phenotype. Fruit firmness was significantly reduced compared with EV fruit (**Figures 7A, B**). Cell wall component analysis showed lower cellulose and hemicellulose contents in VcβGAL369-OE fruit (**Figures 7E, F**). Soluble pectin content increased, whereas insoluble pectin content decreased (**Figures 7H, I**). In addition, cellulase and PG activities were elevated after *VcβGAL369* overexpression (**Figures 7G, J**). These changes were opposite to those observed in the silencing assay, supporting a positive role for *VcβGAL369* in cell wall disassembly and blueberry fruit softening.

## 4 Discussion

### 4.1 Structural and physiological features associated with the firm-fruited genotype

Fruit firmness is a major determinant of blueberry postharvest quality because softening reduces storability, transportability, and commercial value. Previous studies have linked blueberry softening with changes in cell wall composition, polysaccharide organization, and the activities of enzymes involved in cell wall modification (Chen et al., 2015; Ji et al., 2021; Trandel-Hayse et al., 2023). In the present study, ‘Duke’ and the firm-fruited genotype ‘2017D’ showed pronounced differences in texture throughout fruit development. These differences were evident not only in firmness but also in several TPA-derived parameters, including chewiness, gumminess, resilience, springiness and cohesiveness. The coordinated changes in these traits indicate that the greater firmness of ‘2017D’ was accompanied by broader differences in fruit textural properties rather than variation in a single texture index.

Changes in soluble solids and titratable acidity further revealed differences in physicochemical characteristics between the two genotypes during fruit development. Such differences may reflect variation in ripening progression together with inherent differences in fruit physiology between ‘Duke’ and ‘2017D’. Fruit softening is a multifactorial process involving not only cell wall remodeling but also changes in cuticle properties, water relations, turgor, and other ripening-associated processes (Yan and Castellarin, 2022). Therefore, the contrasting texture profiles observed here are likely to reflect the combined effects of multiple structural and physiological processes.

Histological observations were consistent with the contrasting texture profiles. At the green stage, the two genotypes showed broadly similar mesocarp organization, whereas more pronounced tissue disorganization was evident in ‘Duke’ at the blue stage. In contrast, ‘2017D’ retained a comparatively more continuous mesocarp organization. Quantitative morphometric analysis further showed a larger cell-profile area and greater periclinal and anticlinal dimensions in ‘2017D’ at 75 dpa. Similar associations between tissue organization and fruit mechanical properties have been reported in other fleshy fruit systems (An et al., 2023). These observations suggest that differences in mesocarp structural organization accompany the contrasting firmness of ‘Duke’ and ‘2017D’ during ripening.

### 4.2 Cell wall remodeling underlying firmness retention in ‘2017D’

Fruit softening is closely associated with remodeling of the primary cell wall and middle lamella, including pectin solubilization, hemicellulose modification, and changes in cellulose-associated networks (Brummell and Harpster, 2001; Brummell, 2006; Goulao and Oliveira, 2008). In blueberry, firmness variation has also been associated with differences in cell wall polysaccharide composition, calcium-associated wall structure, and hemicellulose content (Olmedo et al., 2021). In the present study, ‘2017D’ generally retained higher cellulose and hemicellulose contents during development and maintained higher insoluble pectin levels, whereas ‘Duke’ accumulated more soluble pectin during the later developmental stages. These patterns are consistent with more extensive cell wall polysaccharide remodeling and pectin solubilization in the softer genotype.

The enzyme activity data further revealed distinct differences in cell wall remodeling between the two genotypes. PG activity was lower in ‘2017D’, whereas β-GAL activity was also lower at several developmental stages, particularly during ripening. PG-mediated homogalacturonan depolymerization contributes to pectin disassembly and softening, as demonstrated in strawberry (Quesada et al., 2009; Paniagua et al., 2020). β-GAL primarily removes terminal galactosyl residues from cell wall polysaccharides, including galactan-rich pectic side chains, and down-regulation of strawberry FaβGal4 increased wall galactose levels and reduced fruit softening (Smith and Gross, 2000; Paniagua et al., 2016). The lower PG and β-GAL activities in ‘2017D’ are consistent with its greater retention of insoluble pectin and firmness during ripening.

Although ‘2017D’ exhibited higher cellulase activity at the purple stage, cellulose and hemicellulose contents remained higher than those in ‘Duke’. This suggests that cellulase activity alone did not determine the net retention of structural polysaccharides. Cell wall architecture, substrate accessibility, and continued polysaccharide synthesis may have contributed to the greater retention of cellulose and hemicellulose in ‘2017D’. Consistently, altered endo-β-1,4-glucanase expression in strawberry did not necessarily lead to proportional changes in fruit softening (Palomer et al., 2006). Thus, the greater firmness retention of ‘2017D’ appears to be associated with differences in multiple aspects of cell wall remodeling, rather than a uniform reduction in all activities involved in cell wall modification.

### 4.3 Transcriptomic prioritization of cell wall-related candidate genes

At the blue stage, RNA-seq revealed differences between the two genotypes in pathways related to hormone signaling, carbohydrate metabolism, glycan degradation, phenylpropanoid biosynthesis, and cell wall remodeling. These enriched pathways are consistent with the involvement of ripening physiology and wall metabolism in fruit texture formation (Brummell, 2006). Among the initially screened cell wall-related candidates, several genes encoded proteins with known or predicted roles in wall modification, including EXPs, XTHs, PEs, PGs, PLs, cellulases, cellulose synthases, and β-GALs. These gene families have been implicated in wall loosening, pectin metabolism, hemicellulose remodeling, and fruit texture changes in different species (Brummell and Harpster, 2001; Su et al., 2024).

The expression heatmap revealed contrasting expression patterns among the candidate genes, indicating that ‘Duke’ and ‘2017D’ differed in the expression of cell wall-related genes at the blue stage. qRT-PCR analysis across developmental stages further revealed distinct expression patterns among selected candidates across genotypes and developmental stages. Among these genes, *VcβGAL369* showed a pronounced expression difference between the two genotypes in the RNA-seq analysis together with a distinct developmental expression pattern. Its higher expression in ‘Duke’ during ripening was also consistent with the higher β-GAL activity observed in the softer genotype at several developmental stages. These expression and biochemical characteristics, together with its annotation as a putative β-galactosidase involved in cell wall polysaccharide modification, supported the selection of *VcβGAL369* for further structural and functional characterization. Given that the RNA-seq comparison involved two blueberry materials at a single developmental stage, the transcriptomic data were used primarily for candidate prioritization, while broader validation across additional genotypes and developmental stages will be required to determine the generality of the identified expression patterns.

### 4.4 VcβGAL369 contributes to blueberry fruit softening

Transient silencing and overexpression provided reciprocal evidence that *VcβGAL369* is involved in blueberry fruit softening. Silencing of *VcβGAL369* reduced transcript abundance and β-GAL activity, increased fruit firmness, and promoted the retention of cellulose, hemicellulose, and insoluble pectin. In contrast, transient overexpression increased *VcβGAL369* expression and β-GAL activity, reduced firmness, increased soluble pectin, reduced insoluble pectin, and enhanced PG and cellulase activities. These opposite responses support a role for VcβGAL369 in promoting softening-associated cell wall remodeling.

Given the established functions of GH35 β-galactosidases, *VcβGAL369* may contribute to the removal of terminal galactosyl residues from cell wall polysaccharides, particularly galactan-rich pectic side chains. Such modification may alter the organization and accessibility of the surrounding wall matrix to other enzymes involved in cell wall modification (Paniagua et al., 2014). The coordinated changes in β-GAL activity, pectin fractions, other cell wall-related enzyme activities, and fruit firmness following manipulation of *VcβGAL369* are consistent with its involvement in broader cell wall remodeling during fruit softening. However, the downstream interactions between β-GAL-mediated side-chain modification and other wall-remodeling processes remain to be resolved.

Overall, the phenotypic, histological, biochemical, transcriptomic, and transient functional evidence indicates that the greater firmness retention of ‘2017D’ is associated with a comparatively more continuous mesocarp organization, retention of structural cell wall components, reduced pectin solubilization, and differences in cell wall remodeling associated with differences in β-GAL and PG activities. *VcβGAL369* appears to contribute to blueberry fruit softening within this broader remodeling process. These findings highlight *VcβGAL369* as a candidate for further studies on fruit texture regulation and postharvest firmness.

## 5 Conclusion

In summary, this study integrated phenotypic, physiological, histological, biochemical, transcriptomic, and transient functional analyses to investigate the basis of firmness variation between ‘Duke’ and the firm-fruited genotype ‘2017D’. The greater firmness retention of ‘2017D’ was accompanied by a comparatively more continuous mesocarp organization, greater retention of cellulose, hemicellulose, and insoluble pectin, and lower PG and β-GAL activities during ripening. Transcriptomic and qRT-PCR analyses further revealed differences between the two genotypes in the expression of multiple genes associated with cell wall remodeling. Among these candidates, VcβGAL369 showed a pronounced expression difference together with a distinct developmental expression pattern and was therefore selected for functional characterization. Transient silencing and overexpression assays supported a role for *VcβGAL369* in promoting blueberry fruit softening, accompanied by changes in fruit firmness, cell wall components, and related enzyme activities. Together, these findings indicate that the contrasting firmness of ‘2017D’ and ‘Duke’ is associated with differences in cell wall remodeling and provide functional evidence supporting the involvement of *VcβGAL369* in blueberry fruit softening. This study provides a basis for further investigation of the molecular mechanisms underlying firmness regulation and postharvest texture improvement in blueberry.

## Data Availability

The datasets presented in this study can be found in online repositories. The names of the repository/repositories and accession number(s) can be found in the article/**Supplementary Material**.
