# Supplementary material for: Integrated physiological and transcriptomic analyses reveal cell wall remodeling underlying firmness retention in a firm-fruited blueberry genotype
Source: Front Plant Sci. 2026 Oct 8;17:1962192. doi: 10.3389/fpls.2026.1962192 (PMC13686834; doi:10.3389/fpls.2026.1962192)
Supplement: Supplementary file 1 [file SupplementaryFile1.docx]

**Supplementary data for**

**Integrated physiological and transcriptomic analyses reveal cell wall remodeling underlying firmness retention in a firm-fruited blueberry genotype**

**Supplementary material:**

Supplementary Fig. S1. SSR marker-based validation of the genetic relationship between '2017D' and 'Duke'. Amplification products from '2017D' and 'Duke' are shown side by side for each SSR marker. M indicates the 500 bp DNA marker. Among 72 SSR primer pairs, 64 loci showed identical amplification patterns between the two genotypes, whereas 6 loci were polymorphic; primers 26 and 56 showed Duke-specific amplification patterns in '2017D'.

Supplementary Fig. S2. Quantitative morphometric analysis of mesocarp cells in ‘2017D’ and ‘Duke’ at 35 and 75 days post-anthesis (dpa). (A) Cell profile area. (B) Periclinal cell diameter. (C) Anticlinal cell diameter. Individual points represent three independent biological replicates, and diamonds indicate the mean. Error bars represent ± SD. Data were analyzed using linear models including genotype, developmental stage, their interaction, and imaging batch. Genotype contrasts between ‘2017D’ and ‘Duke’ were performed separately at each developmental stage, with *P* values adjusted across the two stage-specific comparisons using the Holm method. Holm-adjusted P values are shown above the corresponding comparisons; ns, not significant.

Supplementary Fig. S3. Phylogenetic relationships and structural characteristics of the VcBGAL gene family in blueberry. The maximum-likelihood phylogenetic tree, conserved motif composition, conserved-domain distribution, and gene structures of 59 VcBGAL family members are shown. VcBGAL1 (*VcβGAL369*) is highlighted in red. Conserved motifs (Motifs 1–10) are represented by different colored boxes, and their positions are shown relative to the corresponding protein sequences. Conserved-domain matches identified by NCBI CDD are displayed according to their positions within each protein; different colors represent BetaGal_dom4_5, Glyco_hydro_35, PLN03059 superfamily, and zf-RVT superfamily hits. Gene structures are shown according to genomic position, with coding sequences (CDS), 5′ untranslated regions (5′ UTRs), and 3′ untranslated regions (3′ UTRs) indicated by blue, green, and yellow boxes, respectively, and introns represented by connecting lines. Protein and genomic positions are indicated by the corresponding scales.

Supplementary Fig. S4. Phylogenetic and structural characterization of *VcβGAL369*. (A) Neighbor-joining tree of *VcβGAL369* and representative GH35 β-galactosidases. AoBGAL from Aspergillus oryzae was used as the outgroup. Bootstrap values from 500 replicates are shown at the nodes. (B) ESMFold-predicted structure of the catalytic region of *VcβGAL369* (aa 24–400), colored according to pLDDT. The putative catalytic residues Glu183 and Glu252 are shown in red. (C) Multiple-sequence alignment of the conserved QIENE and WTEAW catalytic motifs. Glu183 and Glu252 are highlighted in red.
